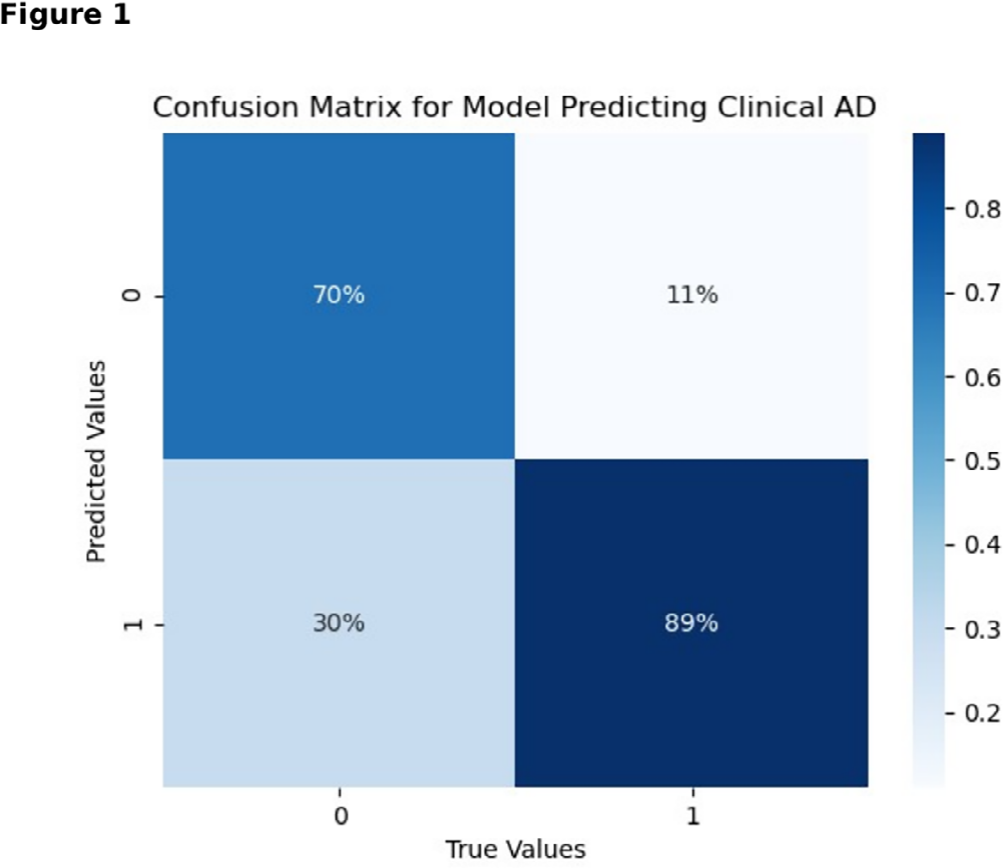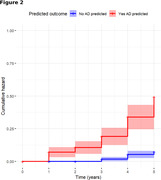# Prediction of Incident Alzheimer's Disease in Older Community‐Dwelling Adults using Wrist Accelerometry and Deep Learning

**DOI:** 10.1002/alz.087666

**Published:** 2025-01-09

**Authors:** Julie Midroni, Lei Yu, Jennifer S Rabin, Xilin Liu, Maged Goubran, Aron S Buchman, David A. Bennett, Andrew Lim

**Affiliations:** ^1^ Temerty Faculty of Medicine, University of Toronto, Toronto, ON Canada; ^2^ Department of Neurological Sciences, Rush University Medical Center, Chicago, IL USA; ^3^ Rush Alzheimer's Disease Center, Rush University Medical Center, Chicago, IL USA; ^4^ Sandra Black Centre for Brain Resilience and Recovery, Sunnybrook Research Institute, Toronto, ON Canada; ^5^ Sunnybrook Research Institute, Toronto, ON Canada; ^6^ Faculty of Engineering, University of Toronto, Toronto, ON Canada; ^7^ University Health Network, Toronto, ON Canada; ^8^ Hurvitz Brain Sciences Research Program, Sunnybrook Research Institute, Toronto, ON Canada; ^9^ Rush University, Chicago, IL USA; ^10^ Sunnybrook Health Sciences Centre, Toronto, ON Canada

## Abstract

**Background:**

Drugs targeting Alzheimer's disease (AD) pathology are likely to be most effective in the presymptomatic stage, where individuals harbor AD pathology but have not manifested symptoms. Neuroimaging approaches can help to identify such individuals, but are costly for population‐wide screening. Cost‐effective screening is needed to identify those who may benefit from neuroimaging, such as those at risk of developing clinical disease. We present a deep learning algorithm that uses accelerometry recordings to predict clinically diagnosed AD in dementia‐free patients.

**Method:**

Participants were from The Memory and Aging Project (MAP), a longitudinal cohort study of older adults focused on aging and dementia. As part of this study, participants were asked to wear a wrist accelerometer for ten days. We designed a feedforward neural network that synthesizes clinical and accelerometric features to predict clinical AD. Clinical features were selected based on ease of collection, and included age, sex, education, social isolation and purpose in life. The dataset included participants without dementia at the time of recording, who survived for and had a known clinical AD outcome within five years of the recording.

**Result:**

The training dataset consisted of 875 unique patients. The test dataset consisted of 395 unique patients (mean age 81.5, SD= 6.8). 14.4% of the test set developed clinical AD within five years. On the test set, the model achieved 89% sensitivity (SN), 70% specificity (SP), and an F1 score of 0.87 (Figure 1). This is superior to accelerometric‐only (SN 67%, SP 70%, F1 0.68) and clinical‐only (SN 76%, SP 80%, F1 0.78) models. Model accuracy was similar for patients both with and without mild cognitive impairment at baseline. When the binarized model output was used as a predictor for five‐year AD‐free survival via Cox proportional hazards (Figure 2), it achieved a C‐index of 0.729 [95% CI 0.69 b‐0.77], a hazard ratio of 6.90 [95% CI 4.15‐11.47], and a log‐likelihood ratio of 71.05. Further tuning and validation of the model are underway.

**Conclusion:**

A deep learning model using wrist accelerometry and easily obtained clinical features shows promise in predicting 5‐year AD outcomes in adults without dementia at baseline.